# The British Medical Supplement

**Published:** 1918-10

**Authors:** 


### THE BRITISH MEDICAL SUPPLEMENT

The British Research Committee, in compiling and reviewing the enemy medical literature, and publishing it each month as the Medical Supplement to the Review of the Foreign Press, is performing a service of real value to the medical officers of the Entente Allies. The Medical Supplement also publishes reviews of important French and Italian articles.

The reviews that appear in the Medical Supplement are of unusual value because they are prepared by specialists in the various branches of medicine and surgery, many of whom are among the most celebrated men in England and Scotland, who not only give the author's viewpoint but often make critical comments upon the articles which they abstract. This is of particular importance in dealing with the enemy literature.

Judging from the reviews or abstracts of German and Austrian medical articles that appear each month in the Medical Supplement even the war has not had a deterring effect upon the prolific pens of Berlin and Vienna doctors; though their publications are of less scientific value than when they had access to the literature of other nations, and could plagiarize the idea and researches of the advanced thinkers and workers in medicine and surgery outside of Germany and Austria. While the statements made by German medical authors must be discounted, because much of what they have claimed as original, both before and since the war began, belongs to the medical men of other nations, it is proper to learn all that we can from them, as well as to give them full credit for their good work. If the Germans or Austrians have made any advances in the treatment of war wounds, or in the prevention and treatment of disease, they should be adopted by our medical men, though what a Boche author says should be verified before it is accepted as a fact.

### Brain Injuries.

In the August number of the Medical Supplement a neat compliment is paid to Lieut.-Col. Harvey Cushing, Senior Consultant in Neurological Surgery of the American Expeditionary Forces. In discussing the German literature on the treatment of injuries of the brain it says: " In this respect the epoch-making work of Harvey Cushing (*Brit. J. Surg.*, Bristol, 1918, 5, 558) is far in advance of anything that has yet appeared both as a clinical record and a practical guide to the surgeon. Similarly, the same author's minute details as to treatment that appeared in the *Brit. M. J.* Lond., 1918, Feb. 23, are of much higher merit than anything hitherto published in enemy countries. So, also, the work of Châtelin and de Martel (*Blessures du Crâne et du Cerveau.* Paris, Masson, 1917) gives a better general review of the whole subject than is to be found in German literature."

Since the Medical Supplement supplies a large number of copies to the American Expeditionary Forces in exchange for *War Medicine*, and since its reviews of the German and Austrian medical journals are so well done, *War Medicine* does not publish abstracts of enemy literature, but confines its abstracts to articles from the French, British, Italian and American journals.

While the Medical Supplement reaches most of the hospitals, and other medical units of the American Expeditionary Forces, if medical officers desire personal copies, they may purchase them (price one shilling net) through any bookseller, or directly from H. M. Majesty Office, Imperial House Kingsmay, London, W. C. 2.

